# The Attachment Doll Play Assessment: Predictive Validity with Concurrent Mother-Child Interaction and Maternal Caregiving Representations

**DOI:** 10.3389/fpsyg.2016.01594

**Published:** 2016-10-18

**Authors:** Carol George, Judith Solomon

**Affiliations:** ^1^Department of Psychology, Mills College, OaklandCA, USA; ^2^School of Clinical Medicine, University of CambridgeCambridge, UK

**Keywords:** assessment, play, attachment, caregiving, representation, synchrony

## Abstract

Attachment is central to the development of children’s regulatory processes. It has been associated with developmental and psychiatric health across the life span, especially emotional and behavioral regulation of negative affect when stressed (Schore, 2001; Schore and Schore, 2008). Assessment of attachment patterns provides a critical frame for understanding emerging developmental competencies and formulating treatment and intervention. Play-based attachment assessments provide access to representational models of attachment, which are regarded in attachment theory as the central organizing mechanisms associated with stability or change (Bowlby, 1969/1982; Bretherton and Munholland, 2008). The Attachment Doll Play Assessment (ADPA, George and Solomon, 1990–2016; Solomon et al., 1995) is a prominent established representational attachment measure for children aged early latency through childhood. This study examines the predictive validity of the ADPA to caregiving accessibility and responsiveness assessed from mother-child interaction and maternal representation. Sixty nine mothers and their 5–7-year-old children participated in this study. Mother-child interaction was observed during a pre-separation dyadic interaction task. Caregiving representations were rated from the Caregiving Interview (George and Solomon, 1988/1993/2005/2007). Child security with mother was associated with positive dyadic interaction and flexibly integrated maternal caregiving representations. Child controlling/disorganized attachments were significantly associated with problematic dyadic interaction and dysregulated-helpless maternal caregiving representations. The clinical implications and the use of the ADPA in clinical and educational settings are discussed.

## Introduction

Attachment has been associated with developmental and psychiatric health across the life span, especially emotional and behavioral regulation of negative affect when stressed (Schore, 2001; Schore and Schore, 2008). The synergy of children’s emotional, social, cognitive, and language development beginning around age 4 years poises play to be a rich and reliable methodology for assessing attachment (Solomon et al., 1995; Bretherton and Munholland, 2008). Attachment theory posits that internal representational models of attachment are derived from real experience with attachment figures. They are modulated and regulated by patterns of defensive exclusion and thus influence procedural scripts, memories, evaluations of self and attachment figures; as such, knowing children’s representational “rules” enable us to understand how they view their world and make predictions about their development (Bretherton, 2005; Waters and Waters, 2006). Representations are the central organizing mechanism associated with stability or change, and provide a critical frame of reference for child treatment and family intervention (Bowlby, 1969/1982, 1980; Hodges and Steele, 2000; Hodges et al., 2003; Hoffman et al., 2006; Oppenheim and Goldsmith, 2007; Bretherton and Munholland, 2008).

Attachment researchers have developed a range of play-based methods to assess children’s attachment patterns in which children “play out” attachment themes. Research findings support theoretically derived predictions regarding the associations between attachment security children’s emotional and behavior adjustment in community and risk samples (Oppenheim, 1997; Verschueren and Marcoens, 1999; Goldwyn et al., 2000; Rydell et al., 2005; Goodman et al., 2007, 2012, 2013; Green et al., 2007; Venet et al., 2007; Bureau and Moss, 2010; Stievenart et al., 2011; Webster and Hackett, 2011; Torres et al., 2012; Miljkovitch et al., 2013; Salari et al., 2016). Research findings also support predictions of a disproportionate amount of attachment insecurity (especially disorganized children) in risk samples (e.g., divorce, maltreatment, adoption, foster care, institutionalized) (Gloger-Tippelt and König, 2007; Katsurada, 2007; Román et al., 2012; Torres et al., 2012; Bovenschen et al., 2016).

Caregiver accessibility and responsiveness to the child is the central explanatory mechanism of attachment security (Bowlby, 1969/1982; Ainsworth et al., 1978). Demonstration of an association between attachment security and the attachment-caregiving relationship is therefore considered to be a core construct, essential to validating any measure of attachment. Such studies are sparse, however (Solomon and George, 2016). The present study examines the validity of the Attachment Doll Play Assessment (ADPA, George and Solomon, 1990–2016; Solomon et al., 1995), reporting on two aspects of this core construct: mother-child interactive synchrony and maternal caregiving representation. We begin with an overview of attachment play-based assessments so as to provide an interpretive frame for this study.

### Attachment Assessments Using Play

The field of attachment uses “doll play” to tap children’s symbolic play around attachment themes. Doll play procedures follow Bretherton’s original approach to studying symbolic representation in the preschool period using the Attachment Story Completion Task (ASCT, Bretherton et al., 1990). The ASCT was originally developed to assess attachment security in 4-year-olds. Over several decades, a range of different protocols have appeared in the literature, most of which follow Bretherton et al.’s (1990) approach. The assessment is administered in a private setting, such as a laboratory or clinical office. The play materials are family dolls and props that create a symbolic “house.” Houses range from using minimal props, such as the ASTC (Bretherton et al., 1990) to an elaborately configured Victorian style doll house (e.g., Green et al., 2000). The procedure used to determine the core play family varies across methods. The most common procedure follows the ASCT, whereby the adult (the researcher or clinician) creates a doll family comprised of two dolls and two children, a doll designated as the self and a sibling of the same gender. By contrast, some researchers ask the children to select the family. The ADPA procedure, for example, instructs the children first select the self and then select other family members (George and Solomon, 1990–2016). The majority of procedures then use the designated family for the entire assessment, with one exception – a procedure that asks children if they wish to reconfigure the family members before each story (Farnfield, 2015).

The administration technique is analogous across procedures. The goal is for the adult interviewer to systematically introduce story topics, termed story stems. Story stem scripts are followed exactly, and the interviewer’s only interaction with children during their play is to use standardized scripted prompts to encourage children to describe and enact what happens in relation to the attachment topics. Topics are typically limited to four to five core themes conceived in attachment theory as activating the need for parental comfort and protection (Bowlby, 1969/1982): parent-child tension, mildly frightening events, parent separation, and parent reunion. A few procedures add extra topics, such as themes related to parental divorce (Page and Bretherton, 2003); others add a set of generalized stressful topics (e.g., parent loses keys; school bully) (Macfie et al., 2014; Farnfield, 2015). Doll play assessments frequently are used to establish attachment classification groups [secure/insecure; four attachment groups (Ainsworth et al., 1978; Cassidy et al., 1987–1992)]. Classification rubrics typically are developed using *a priori* intuitive extrapolation from other attachment measures or statistical composites based on summing rating dimensions (Bretherton et al., 1990; Green et al., 2000; Gloger-Tippelt et al., 2002). A different approach uses classification criteria based on attachment-expert opinions of essential representational elements (Miljkovitch et al., 2003). Some investigators also report the use of scales to augment or in lieu of classification (e.g., sensitivity, aggression, security, discourse coherency) (Green et al., 2000; Macfie et al., 2008; Webster and Hackett, 2011).

Despite procedural differences, the validity of the doll play method is fairly well established. Validity with other standard attachment measures (e.g., Strange Situation, Attachment Q-Sort) ranges from excellent to acceptable, depending on the method and if measurements were concurrent or administered at different ages. Associations tend to be strongest for comparisons of secure versus insecure attachment groups, for security scales, and associations with attachment disorganization (see Solomon and George, 2016). These procedures are reported to be valid for the use with children ages 3–12 years, although caution should be used when interpreting the doll play assessments of children under 4 years (R. S. Marvin, personal communication, November, 8, 2014). Doll play assessment has been used with English and non-English speaking children (Canada, France, Germany, Israel, Japan, Spain) (Solomon et al., 1995; Goodman and Pfeffer, 1998; Goldwyn et al., 2000; Green et al., 2000; Gloger-Tippelt et al., 2002; Verschueren et al., 2006; Yamakawa, 2006; Katsurada, 2007; Dubois-Comtois and Moss, 2008; Dubois-Comtois et al., 2011; Román et al., 2012; Goodman et al., 2013; Miljkovitch et al., 2013; Farnfield, 2015; Bovenschen et al., 2016).

#### The Attachment Doll Play Procedure (ADPA, George and Solomon, 1990–2016; Solomon et al., 1995)

The ADPA is a doll play attachment assessment that follows the tradition of the ASCT (Bretherton et al., 1990). It uses the same basic attachment story stems: *Hurt Knee* (child’s knee is hurt by falling off a rock), *Monster in the Bedroom* (child, when sent to bed, tells the parents that there is a monster in the bedroom), *Separation* (parents provide a babysitter to stay at home while they go on an overnight trip), and *Reunion* (parents return from their overnight trip).

There are several key elements in the procedure that differentiate the ADPA from the ASCT and other doll play procedures described in the literature. The most important of these is the classification scheme. The ADPA scheme is based on Bowlby’s (1973, 1980) description of defensive processes related to separation and loss (George and Solomon, 1990–2016; George and Solomon, 1998, unpublished) and can also be detected in mothers’ internal caregiving representations and in adult responses to free-response (“projective”) attachment stimuli (Solomon and George, 1999b; George and West, 2011, 2012). Security is conceived in terms of the flexible integration of attachment-related thoughts and feelings, whereas strategies of defensive exclusion of information can be systematically brought into play as responses to anxiety regarding attachment figures. These processes include “deactivation” (prevention of attachment-related thoughts and feelings), associated with avoidant classifications, and “cognitive disconnection” (disconnection from awareness of the links between affect and thought), associated with ambivalent classifications. When attachment-related distress cannot be contained (assuaged), “dysregulation” of the attachment system (or, in Bowlby’s 1980, terms, a “segregated system”) is likely to be the result. Depiction of uncontained frightening and catastrophic events, as well as persistent constriction (refusal to play), are the single most defining indices of dysregulation and attachment disorganization (Solomon et al., 1995). The ADPA has demonstrated construct validity; there is significant concordance between the representation classifications and attachment classifications based on children’s reunion behavior with the parent (Solomon and George, 2002; Yamakawa, 2006; Dubois-Comtois et al., 2011).

Concurrent evaluation of ADPA classifications demonstrated that the children’s responses to the combination of the *Separation* and *Reunion* stories evaluated best predicted children’s reunion classification (Solomon et al., 1995). Responses to other stories were more weakly associated with reunion classifications, suggesting that classification schemes that combine all story responses in an additive way are likely to introduce classification error (Solomon and George, 2002).

The ADPA offers some advantages over other doll play systems. The concurrent validation for the four-group classification system that is the most prominent rubric to evaluate attachment patterns in the field provides confidence in the ADPA classification that is not available for other methods, such as the ASCT for example. The MCAST does not differentiate among insecure classification patterns. Most other methods assess doll play in terms of security score (secure vs. insecure), and do not provide classification information (see Solomon and George, 2016 for overview). The examination of doll play against parent reunion also clarified that classification is not an additive process, a finding that is consistent with attachment theory. Attachment is activated differently in different children. Children who are secure in the Strange Situation, for example, may look like avoidant children if only observed during the first reunion with their attachment figure (Ainsworth et al., 1978). These children are thought to need more of a “push” so to speak to demonstrate the secure pattern and classification depends heavily on the observation of the second reunion (Ainsworth et al., 1978). In the same vein, we have noted the secure children, for example, develop stories in response to *Hurt Knee* or *Monster in the Bedroom* that would be associated with children with insecure classifications. Yet their *Separation-Reunion* sequence is the story material that fits their reunion with their mother (Solomon and George, 2002). For example, some children demonstrate sturdy independence in response to the injured knee, and tell a story that would be evaluated as avoidant. We also observed that separation stories do not differentiate among the stories of children in different attachment groups. In short, as in the Strange Situation, it is the representational reunion that provides the best classification information, and it remains an empirical question as to how to think about children’s responses to the other story stems (Solomon and George, 2002).

The ADPA has been demonstrated to predict a range of theoretically expected variables in normative and high-risk samples (Dubois-Comtois and Moss, 2008; Stacks and Oshio, 2009; Bureau and Moss, 2010; Dubois-Comtois et al., 2011; Goodman et al., 2013; Salari et al., 2016).

### The Association between Attachment Doll Play Assessments and Maternal Caregiving

As previously noted, the association between the children’s attachment and parental caregiving is a core tenet of attachment theory. Yet there are only three published studies using attachment doll play procedures that report on these associations. All of these studies are of French-speaking children.

Two reports are from Moss et al. (1997) longitudinal study of attachment beginning in the preschool years. Dubois-Comtois and Moss (2008) and Dubois-Comtois et al. (2011) reported significant associations between children’s attachment doll play representations and concurrent mother-child interactive behavior in a sample heterogeneous as to family background, income, including head of household, and education. Children’s attachment classifications were assessed at age 8.5 years using the ADPA. Mother-child interaction and mother-child conversation were observed in two settings, 3 years earlier during snack time in the laboratory and concurrent assessment of family interaction. The results were similar for mother-child interaction at 5.5 years and family interaction at 8.5 years. The interactions of secure children were more coherent and reciprocal than the interactions of disorganized children, with patterns falling in the middle for avoidant and ambivalent-resistant children. Logistic regression results showed that concurrent interaction (family interaction) was a more powerful predictor, however, than mother-child interaction at age 5.5 years. Dubois-Comtois et al. (2011) also reported a significant association between children’s representational classification at 8.5 years and mother-child conversation during snack time at age 5.5 years.

Miljkovitch et al. (2013) used the ACST to assess doll play representation in a sample of 3½-year-old children who were preterm and full term infants. The study goal was to examine associations between doll play representations and mother interaction. The study results showed significant associations between difficult and problematic mother-child interactions and disorganized attachment for the full-term children, but not for the preterm children. Although these results are consistent with previous research on attachment disorganization, conclusions about the association between doll play assessments and mother-child interaction may be constrained by infant development (Sameroff, 1993) and the age when the ACST was administered.

### The Current Study

This study is the first to examine the concurrent associations between the attachment doll play classifications and parenting. The first set of hypotheses pertained to the association between ADPA attachment classifications and mother-child interaction. Interactions with dyads with secure children were expected to more balanced and harmonious than interaction in dyads with insecure children. Interaction in dyads with disorganized children was expected to be the least balanced and harmonious.

Two other sets of hypotheses examined the role of maternal caregiving representations as related to both ADPA attachment classifications and mother-child interaction. The attachment theory view of caregiving posits that it is guided by the caregiving behavioral system, following Bowlby (1969/1982) ethological model of attachment. Caregiving system processes regulate representations of self, child, and evaluations of their relationship that are consolidated over time based on experiences with the child (George and Solomon, 1996, 2008; Solomon and George, 1996). Representations are conceived as reflecting mother-child interaction (Solomon and George, 1996; George and Solomon, 2008).

The evaluation of caregiving representation in this study is the same as was described for the ADPA. This approach follows Bowlby’s (1980) model of defensive exclusion (see George and Solomon, 2008), defined as unconscious and automatic sorting and exclusion processes that guide and organize representation and behavior. When defensive processes are flexible and integrated, caregiving representations emphasize flexibility, synchrony, adjustment, and mutual enjoyment – qualities associated with maternal sensitivity and children’s attachment security (Pasco Fearon and Belsky, 2016). Flexible integration supports secure base behavior and children’s competence, and differentiates between mothers of children with secure and insecure attachment (George and Solomon, 1989, 1996, 2008; Solomon and George, 1999a). By contrast, when defenses are dysregulated, caregiving is at least to some degree disabled and the caregiving-attachment relationship may be said to be dysregulated as well (Solomon and George, 1996, 2011a; George and Solomon, 2008). In these cases, mothers are likely to be overwhelmed by their worst fears about self and child and report becoming flooded by feelings of being out of control, and vulnerable. In essence, these mothers are rendered *helpless* to care for and protect their children (George and Solomon, 2008). *Dysregulated-helplessness* is the term, we use for this dimension of defensive processing (George and Solomon, 2008; Solomon and George, 2011a). This dimension differentiates between mothers of children with disorganized/controlling and organized attachments (secure, avoidant, ambivalent-resistant) and has been shown to be positively associated with parental stress and child behavior problems in infancy and childhood (George and Solomon, 1996, 2008, 2011).

There is considerable support in the attachment literature for the association between mothers’ mental processes (e.g., mentalization, reflective functioning) and dyadic interaction (see George and Solomon, 2008, for a complete discussion). This is the first study, however, to examine these associations from the perspective of the caregiving system and the caregiver’s representations.

It was hypothesized that there would significant associations between maternal caregiving representations and children’s representations of attachment and mother-child interaction. The mothers of children judged secure using the ADPA were expected to have significantly higher flexible integration ratings than mothers of insecure children; and mothers of disorganized-controlling children were expected to have significantly higher dysregulation-helplessness ratings than mothers of organized children (secure, avoidant, ambivalent). Similarly, flexibly integrated caregiving representation ratings were expected to be positively associated with balanced and harmonious mother-child interaction; and dysregulated-helpless caregiving representation ratings were expected to be inversely associated with balanced and harmonious interaction ratings, thus indicative of interaction problems.

## Materials and Methods

### Participants

The participants were 69 mother-child dyads recruited through private and public kindergarten classrooms in the San Francisco Bay Area (Solomon et al., 1995). School principals and directors were first provided letters describing the study, following the guidelines of the institutions’ internal review boards or school districts. All of the schools that were approached approved the study, and similar letters describing the study accompanied by administrators’ support of the study were sent home in children’s classroom packets. Families who were interested in participating returned the letter accompanied by contact information to classroom teachers. The researcher retrieved this information subsequently from the school and contacted families directly by telephone. The study was once again described, and mothers were provided an opportunity during that conversation to ask questions. The only inclusion criteria for schools and classrooms were that they served typically developing children and were within a 15 mile radius of the playroom to make it feasible for dyads to participate without undue travel.

The children (37 girls, 32 boys) were 6 years old (*M* = 68.3 months; range 57–85 months). Thirty five percent of the children were first born, 42% later born, and 23% were only children. Mothers’ mean age was 37.4 years (range 21–49 years). Eight-one percent of the children were living with both parents, 4.3% were living with their mothers in a blended family, and 13% were living with a single head of household mother. Mothers were predominantly college educated (69.6%), Caucasian (80%), with moderate to high incomes.

### Measures

#### Attachment

The child’s attachment classification was assessed using the ADPA (George and Solomon 1990–2016; Solomon et al., 1995). The doll play is administered to each child individually. The doll house is designed as a “single story,” with furniture arranged on a large wooden board to designate the kitchen, living room, bedrooms (child and parent), and the backyard. In addition to basic furniture, props include kitchen and food items, children’s toys, and a few household items (e.g., telephone). The adult interviewer asks the child to first select the doll to be the self and then select the other pretend family members from three sets of culturally diverse dolls (Caucasian, African American, and Asian). Each set includes a mother, father, female child, male child, and baby. The child is asked to put their pretend family in the house and play for 5 min as a warm up. Children are first asked to select the doll they want to be the self, and are then instructed to select the other dolls in their “pretend” family. They are never asked to select family members to represent real family members. Further, there is no requirements for doll selections that fill actual family roles, such as selecting a parent doll. Children are free to select a self as represented by a child or adult doll. As a result, it is not unusual when given this choice in family member selection for children to create families that have no mother or father or to select adult dolls to be the self. Indeed, these selection elements have been shown to be an index of attachment dysregulation and are prominent in children with disorganized attachments (George and Solomon, 1998, Unpublished).

The assessment is comprised of a set of story stems. Once a story stem is introduced, the interviewer asks the child to *“show me what happens next.”* The first story, *Pets*, introduces animals into the play; the mother asks the family if they want to keep the puppy and kitten that have appeared at their door. The next story stems are: *Hurt Knee*, the child falls off of a high rock in the backyard and hurts their knee and can calls out to parents; *Monster in the Bedroom*, the parents tell the child to go to bed and the child cries out that there is a monster in the bedroom; *Separation*, the parents leave to go on an overnight trip and a babysitter stays with the children; and *Reunion*, the parents return the next day.

The attachment classification rubric designates five attachment groups based on children’s responses to the combined evaluation of the *Separation* and *Reunion* stories (Solomon et al., 1995). Coding is done using verbatim transcripts that records the child’s narration and actions. Children judged secure (B) demonstrate family or personal *integration*. Dangers or negative events (e.g., robbers come to the house) are resolved; parents are portrayed as committed and caring; reunions are complete and uninterrupted; or children demonstrate constructive agency during in the parents’ absence. Children judged avoidant (A) demonstrate defensive deactivation. Stories include themes that describe complete shifts in attention that neutralize reunion distress, such as blocking the separation or family members asleep on reunion. Characters act non-chalant and casual. Children judged ambivalent (C) demonstrate defensive cognitive disconnection. Reunions are interrupted or incomplete. Ambivalent children often become mentally busy with small details (e.g., arranging dishes, sweeping the house). Children judged disorganized/controlling (D) demonstrate segregated systems processes that overwhelm other fears of their play, evidencing their underlying attachment fears. There are two forms of disorganized-controlling responses. Controlling punitive (D1) children respond with stories in which themes are frightening and uncontained. Characters are threatened or threatening, helpless, out of control, and the self and family are left at risk of *disintegration.* Controlling caregiving (D2) children are constricted. These children appear to be inhibited and extremely uncomfortable with the doll play task, frequently responding to prompts with only statements such as “I don’t know,” or “nothing happens.” Solomon et al. (1995) showed that this was not due to an inability to play or refusal to play with dolls, and that this response pattern is not a form of avoidance. These children had no problem playing with family like toys (e.g., pretend people in a toy castle or space station) during the free play period. The association between constricted responses during assessment with attachment dysregulation has been confirmed in adult assessment using free response picture stimuli depicting people in attachment situations (George and West, 2012).

The ADPA attachment classification distribution was 16 B’s, 17 A’s, 18 C’s, 18 D’s (13 D1’s; 5D2’s). Classifications were completed by the first and second author. Inter-coder reliability, coded blind for the entire sample 71% (kappa = 0.62), with the highest agreement for disorganized versus organized (secure, avoidant, ambivalent combined) classifications, 95% (kappa = 0.85) (Solomon et al., 1995).

#### Mother-Child Interaction

Dyadic interaction was assessed using Moss et al. (1998) and Moss and St-Laurent (2001) scales for mother-child interaction affective quality, comprised of nine 7-point bipolar rating scales. One scale assesses the *overall* quality of interaction. A high *overall* score reflects balanced and harmonious interaction, with low scores indicating indifference or conflictual interaction. Eight additional subscales assess the components of balanced and harmonious interaction: *coordination* (smooth goal-oriented vs. unproductive flow of interaction), *communication* (verbal and non-verbal clarity vs. inconsistent or incongruous interchange), *partner roles* (appropriate parent-child role assumption vs. role reversal), *emotional expression* (balanced and shared positive and negative affective states vs. imbalanced, negative, or exaggerated expression), *responsivity/sensitivity* (interaction attunement vs. intrusiveness or ignoring), *tension/relaxation* (calm and comfortable vs. tense or anxious), *mood* (generally positive vs. negative), and *enjoyment* (sustained warmth and pleasure vs. displeasure). A principal-components factor analysis of the nine scales yielded a single factor explaining 83% of the variance (Moss and St-Laurent, 2001). Thus, only the overall scale score representing reciprocal, balanced, and harmonious interaction is used in data analysis (following Moss and St-Laurent, 2001).

Raters blind to all participant information rated dyadic interaction from videotape of mother and child “reading” a wordless story book. Rating and reliability were completed in two steps. Fifty one cases were rated by a reliable rater from the Moss lab. Inter-rater reliability between this rater and another trained rater from the Moss lab on 30% of these cases was 81% for the overall rating and ranged from 70 to 88% on the eight subscales. A third rater from our laboratory was trained to 80% reliability on this set of 51 cases and then rated the remaining 18 cases.

Scale validity has been established for children ages 3–7 years old. These scales distinguish mother-child interaction patterns associated with children’s attachment patterns in cross-sectional and longitudinal studies, and have also demonstrated associations with behavior problem ratings and school performance (Moss et al., 1998, 2004a,b; Moss and St-Laurent, 2001). A correlation of 0.71 between the overall rating evaluated in the laboratory and the home observations demonstrated ecological validity for this measure (Dubois-Comtois and Moss, 2008).

#### Maternal Representation of Caregiving

Caregiving representation was assessed using the *Caregiving Interview*, an interview adapted by George and Solomon (1988/1993/2005/2007) from the *Parent Development Interview* (Aber et al., unpublished). The *Caregiving Interview* is a clinical-style interview designed to activate the caregiving system. The interview encourages mothers to describe memories of specific events, interactions, feelings, and their evaluations of these events using a series of open-ended questions about emotions associated with being a parent (e.g., joy, worry, guilt, confidence). Mothers are also asked to describe attachment-related events such as separations and, for this age of children, beginning school. The interview is relationship specific; that is, mothers are asked to focus on specific experiences and their relationship with a particular child rather than describing generic parenting situations.

This study focused on caregiving representations associated with attachment security and disorganization, utilizing two rating scales, *flexible integration* and *dysregulation-helplessness* (George and Solomon, 1988/1993/2005/2007). *Flexible integration* is characterized by descriptions of caregiving flexibility and self-other balance. High scores reflect an age appropriate commitment to the child’s needs, neither at the expense nor indulgence of the mother’s own needs; a capacity to support the child’s competence and autonomy; sincere mutual enjoyment the desire and ability to seek repair relationship in response to tensions or ruptures and the desire to protect and buffer the child from unnecessary distress or risk. *Dysregulation-helplessness* is characterized by descriptions of behavioral and often also representational dysregulation. Situational examples and appraisals consistently demonstrate that attachment-caregiving situations are overwhelming, frightening, or out of control; mothers describe being helpless to find solutions to common childrearing challenges. These examples often also include descriptions of taking extreme measures, including prolonged and angry mother-child confrontations and battles of wills, freezing and being unable to take action, or failure to recognize children’s vulnerability.

Rating is done from verbatim descriptions extracted from the interview of mother-child interaction, called biographical vignettes. All vignettes from a single case are combined into a single transcript and ratings are based on the overall evaluation of these vignettes using 7-point rating scales. High ratings (5–7) are assigned when the scale dimension is predominant. A midpoint rating (4) is assigned when there is clear evidence of that dimension but is not predominant. Ratings 3 and below are assigned when evidence is minimal to absent.

Two sets of raters blind to all information about the participants separately rated flexible integration and helplessness. The audio quality for six interviews was not of sufficient to transcribe, resulting in 63 interviews in the Caregiving Interview sample. Pearson correlations on 20% of the cases demonstrated interrater reliability that ranged from 0.79 to 0.90. Reliability checks between the raters and the first author on these same cases ranged from 0.78 to 0.85. The authors also rated dysregulation-helplessness using interviews for which case identifications were blinded. Correlations between the authors’ ratings and those of trained raters on reliability sets from this sample and other samples ranged from 0.80 to 0.92.

These rating scales have been shown to distinguish among child attachment groups (George and Solomon, 1996, 2008). High ratings for flexible integration and dysregulation-helplessness were, respectively, associated with mothers of secure and disorganized/controlling child attachment (Solomon and George, 1999a, 2011b; George and Solomon, 2008). Flexible integration was associated with attachment security for high partner conflict divorced mothers (Solomon and George, 1999a). A significant positive association between dysregulation-helplessness was found for parenting stress and children’s adjustment problems (George and Solomon, 2011).

### Procedure

Dyads participated in a 90-min laboratory play room laboratory session. After signing consent forms, the dyad was introduced to the laboratory playroom. They were asked to select a book to “read” together from Mayer’s (1976) *Four Frogs in a Box* “wordless” story books. When the story was completed, the mother was escorted to an adjacent room where she was given the *Caregiving Interview*. The child remained in the playroom with a female adult stranger (child interviewer) who administered the ADPA and remained in the room while the child engaged in free play until the mother returned approximately 1 h later.

## Results

### Preliminary Analyses

Pearson correlations among family demographic variables showed significant associations between maternal education and family income (*r* = 0.30, *p* < 0.05), and between marital status and family income (*r* = -0.42, *p* < 0.001). A composite score representing family configuration (i.e., maternal education, family income, marital status) was computed using mean standardized scores for these variables (Moss et al., 2004b). There were no significant associations between family configuration, birth order, and child age and the any of variables of interest. There was a significant association between gender and child attachment classification [*r*(67) = 0.28, *p* < 0.05] and maternal dysregulation-helplessness ratings [*t*(61) = 2.71, *p* < 0.01]. Mothers of boys were rated significantly more helpless than mothers of girls. There were significantly more boys in the disorganized/controlling classification groups than girls (see **Table 1**). Child gender was used as a covariate in analyses related to these variables. There were no significant differences on any study variables between the two disorganized-controlling subgroups. The results from these subgroups were combined for the purpose of analyses.

**Table 1:** Gender distribution of ADPA child attachment classifications.

Classification	Girls	Boys	Total
B	10	7	17
A	9	6	15
C	14	5	19
D1	4	7	11
D2	0	7	7
Total	37	32	69

### Children’s ADPA Attachment Classification and Mother-Child Interaction

The first hypothesis addressed the associations between children’s ADPA classifications and mother-child interaction. The means and standard deviations for all interaction scales are shown in **Table 2**. Only the overall rating was used for analysis; the means and standard deviations for all the interaction subscales are provided for descriptive purposes (following Moss et al., 1998; Moss and St-Laurent, 2001). As predicted, secure dyads demonstrated the highest levels of overall balanced and harmonious interaction and controlling-punitive dyads the least.

**Table 2:** Attachment Doll Play Assessment child attachment classification and mother-child interaction: means.

	Attachment classification				
Mother-child interaction	B (*n* = 16)	A (*n* = 17)	C (*n* = 18)	D1 (*n* = 13)	D2 (*n* = 5)
Overall	4.50	3.35	3.50	2.46	2.80
Coordination	4.38	3.24	3.44	2.38	2.60
Communication	4.44	3.29	3.33	2.96	3.60
Appropriate role assumption	4.25	3.12	3.33	2.23	3.40
Emotional expression	4.44	3.12	3.30	2.77	3.40
Responsivity/sensitivity	4.31	3.12	3.56	2.31	2.80
Tension/relaxation	4.00	2.94	3.11	2.54	3.00
Mood	4.50	3.41	3.56	2.85	3.40
Enjoyment	4.50	3.41	3.78	2.92	3.40

Analysis of covariance (ANCOVA, gender included as a covariate) showed a significant main effect for attachment [F(4,65) = 7.02, *p* < 0.001, partial η^2^ = 0.30]. *T-*tests examining secure attachment dyads in relation to insecure dyads showed significant differences between secure and all insecure groups. Secure dyadic interaction was significantly more balanced and harmonious than the interaction in avoidant [*t*(31) = 2.71, *p* < 0.01], ambivalent [*t*(31) = 2.65, *p* < 0.01], and disorganized [*t*(32) = 6.22, *p* < 0.001].

### Children’s ADPA Attachment Classification and Maternal Caregiving Representation

The second set of hypotheses addressed the associations between children’s attachment classifications assessed using the ADPA and maternal caregiving representation. The means and standard deviations for the caregiving representation rating scales for mothers of children in each attachment group are shown in **Table 3**. The results supported the predicted associations for both flexible integration and dysregulation-helplessness ratings.

**Table 3:** Attachment Doll Play Assessment child attachment classification and maternal caregiving representation: means.

	Child attachment				
Caregiving representation rating	B (*n* = 15)	A (*n* = 15)	C (*n* = 17)	D1 (*n* = 12)	D2 (*n* = 4)
Flexible integration	5.37	2.13	2.24	1.87	2.50
Dysregulation-helplessness	3.00	3.03	2.53	5.83	5.87

MANCOVA results (gender included as a covariate) demonstrated a significant main effect for flexible integration [F(3,60) = 37.16, *p* < 0.001, partial η^2^ = 0.65] and dysregulation-helplessness [F(3,60) = 30.74, *p* < 0.001, partial η^2^ = 0.66]. *T-*tests between secure attachment dyads and the insecure dyads showed significant differences between secure and all insecure groups. The flexible integration ratings for mothers of secure children were significantly greater than the mothers of avoidant [*t*(28) = 9.02, *p* < 0.001], ambivalent [*t*(30) = 8.39, *p* < 0.001], and disorganized [*t*(29) = 9.90, *p* < 0.001] dyads.

*T-*tests between disorganized attachment dyads and organized dyads also showed significant differences between groups. The dysregulation-helplessness ratings for mothers of disorganized children were significantly greater than the mothers of secure [*t*(29) = 8.22, *p* < 0.001], avoidant [*t*(29) = 10.48, *p* < 0.001], and ambivalent [*t*(29) = 12.46, *p* < 0.001] dyads. There were no significant differences in dysregulation-helplessness ratings among the mothers of children with organized attachments.

### Associations between Mother-Child Interaction and Maternal Caregiving Representation

The final set of hypotheses addressed the associations between mother-child interaction and maternal caregiving representation. Analyses using two-tailed Pearson correlations supported both hypotheses. Representational flexible integration was positively associated with balanced harmonious mother-child interaction [*r*(63) = 0.49, *p* < 0.001]. Representational dysregulation-helplessness was inversely associated with balanced harmonious interaction [*r*(63) = -0.38, *p <* 0.01].

## Discussion

This study was the first to examine the concurrent predictive validity of the ADPA and dimensions assessing mother-child interactive behavior and maternal representations of caregiving. Further, this study adds significantly to what is a sparse literature examining any doll play procedure in relation to core attachment theory constructs.

As predicted, the findings demonstrated that secure mother-child dyads, classified using the ADPA, engaged in significantly greater balanced and harmonious interactions than insecure dyads, with disorganized-controlling dyads showing the most interactive problems. This finding is consistent with longitudinal studies in which parent-child interaction and the ADPA doll play classification were evaluated several years apart (Dubois-Comtois and Moss, 2008; Dubois-Comtois et al., 2011). This finding is also consistent with the one other study of this kind, using the ACST (Miljkovitch et al., 2013) and studies demonstrating associations between mother-child interaction and attachment assessed using reunion procedures (e.g., Humber and Moss, 2005). Further, the results of the current study echo observations of the dyadic interaction breakdown that has been shown characteristic of disorganized toddlers under stress (Solomon and George, 1999a) and are consistent with a robust literature documenting mother-child interaction problems for disorganized and controlling children (Lyons-Ruth and Jacobvitz, 2016).

This study also found strong significant associations between mothers’ caregiving system representations and children’s attachment patterns. As predicted, mothers of secure children were differentiated from mothers of insecure children on the dimension of representational flexibility and integration. The interviews of mothers of secure children were characterized by descriptions of events with their children that expressed trust, cooperation, knowledge of self and child as individuals, and the joy of parenting. They described communicating clearly about caregiving and attachment goals, and thinking about a balanced solution when parents’ and children’s goals conflicted. These representational qualities contribute to sensitivity and the robust association in attachment literature between a range of “sensitive” parenting mental states and attachment security (see George and Solomon, 2008 for a literature review).

By contrast, ratings for dysregulated-helpless caregiving were highest in mothers of disorganized-controlling children. Representations of caregiving in these mothers were markedly out of balance, consistent with the controlling nature of their children on reunion (Solomon et al., 1995). Descriptions of events revealed the clear potential for *failed* protection, a phenomenon we have termed “abdication” of care (Solomon and George, 1996; George and Solomon, 2008). The descriptive vignettes of mothers of disorganized-controlling children mirrored their children’s doll play stories. For mothers of punitive children, care and conflict with their children unleashed their worst fears. Mothers described themselves as being as out of control (e.g., acting like “maniacs,” defiant, hysterical, threatening), and their children were often cast as antagonists, “devils” that rendered them helpless to combat or organize their children’s behavior. Mothers of caregiving children often described themselves as psychologically or behaviorally frozen. They also had difficulty describing mother-child interaction vignettes, and could abruptly stop speaking in mid-sentence. They often described the need for absolute withdrawal from interacting with their children, sometimes associated with descriptions of becoming frightened that they could not maintain behavioral or emotional control if they remained in these situations. Their children tended to be described in the role of parental care, “angels” who did no wrong and were compassionate and sensitive not only to their mothers but had remarkable empathy for all living creatures. Lacking even a basic sense of “felt protection,” it is no wonder that the doll play of punitive and caregiving children was, respectively, wild and out of control or frozen and constricted.

The results of this study also add to the literature demonstrating significant associations between mothers’ representational states of mind and mother-child interaction for mothers of secure children. These include, for example, studies examining representational sensitivity and mind mindedness (e.g., Oppenheim and Koren-Karie, 2002; Grienenberger et al., 2005; Demers et al., 2010). There are no other studies to date that have examined the relation between maternal representation and mother-child interaction for disorganized-controlling children.

This study failed to find differences in any variables for the disorganized-controlling subgroups (punitive, caregiving). There were no differences in mother-child interaction between these two groups of children, suggesting that these mothers equally communicate a sense of failed protection and abandonment to their children. This finding stands in contrast, however, to the results reported by studies by Moss et al. (2004a,b), who found interaction differences between the D1 and D2 groups in both the preschool and childhood years. One explanation for the failure to find differences between the dis-organized-controlling groups in this study may stem from the disproportionate number of boys with disorganized-controlling attachments in the current sample. Seventy four percent of the children in the disorganized/controlling group in the current study were boys, as were all of the children in the controlling-caregiving subgroup. Mothers of boys reported significantly higher helplessness ratings than mothers of girls. Although gender differences are not predicted by attachment theory, research is beginning to demonstrate that gender predicts divergent interactive behavior and child development outcomes as children grow beyond infancy (Hazen et al., 2011; Pasco Fearon and Belsky, 2016), with boys being more difficult than girls.

In addition to the gender composition of the disorganized-controlling attachment group, there are several other limitations to the current study that should inform future research. With regard to the ADPA, studies are needed to investigate test-retest reliability. Further, future research would benefit from including samples with children younger and older than this sample. Finally, the cross-sectional concurrent design cannot address questions of continuity and discontinuity in the associations reported here.

### Clinical Importance of the ADPA

The inspiration to use semi-structured doll play to assess children’s internal working models of their attachment relationships drew on the tradition of child psychotherapy, pioneered by early clinicians such as Anna Freud and Melanie Klein. The results of this study permit the field of attachment to repay that debt. First, it provides empirical validation for the clinical insight that such play is indeed a window into the child’s inner world and it is indeed related to the observed quality of interaction patterns with attachment figures. Second, it demonstrates that the quality of the children’s attachment to their mothers provides the framework for the *organization* of that play. Hence, it is advantageous to clinicians and educators to have a thorough understanding of attachment theory and of attachment-related defenses in order to make sense of the doll play of any particular child.

Variations in doll play associated with each of the attachment groups provide useful information about what is normative and what is potentially problematic. Most striking is the fact that similar “florid” symbolic content can be typical of both secure and disorganized children. Many secure children in this study created disaster scenarios in response to story probes, leading the naïve observer might feel concern about children’s emotional regulation capacities or experiences. In fact, the rich narratives of secure children probably indicate an ability to express and flexibly integrate normative fears of separation and loss. Significantly, the failure to depict the successful resolution of their fears was the distinguishing feature of children in the disorganized group. Similarly, the placid stories or the party themes of some insecure children are likely perhaps to falsely reassure observers just as they probably do the children themselves. These stories, in fact, belie the significant anxieties of these children and the constraints they feel to express or experience them.

In a similar vein, it is important for observers to be aware that the content of doll play is indeed symbolic and strongly influenced by fantasy and associative processes. The children in this study—even the disorganized/controlling children—did not literally experience airplanes flying in the house, volcanoes, witches, getting lost and the like; probably none of them had in this sample experienced maltreatment in a formal sense. When doll play is observed among children who are clinically referred for behavior problems, clinicians in training or untrained observers have a tendency to take story content literally. Indeed, we have known observers to wish to make social services reports on the basis of the play or actually to do so. Just as the interpretation of dreams requires patient analysis of dream content based on knowledge of or interaction with an individual, we caution those new to this kind of work to maintain an open-minded stance regarding doll play assessments. Play content is best used to formulate hypotheses about the child’s experience that can be confirmed or clarified over time.

In fact, if we view the doll play as essentially indicative of children’s defenses, there is all the more reason for educators and observers to adhere to the usual unobtrusive stance of experienced play therapists, who refrain from interpretation or challenging children’s defenses until a therapeutic alliance is well-established. The same should be said about children’s failures to engage in symbolic play during the ADPA. “I don’t know” in response to continued probes does not necessarily mean that task administration of the task has failed in some way or that children lack knowledge or desire to be difficult (though these may sometimes be the case). A more productive assumption is that these children are fearful of entertaining their own thoughts and emotions; such defenses require more, rather than less respect.

Access to the results of attachment security assessments *at a general level* can expand clinicians’ and educators’ understanding of children in their care by providing information about children’s representation of self in relationships. Direct observation of children’s doll play on attachment themes may help to clarify the emotional underpinnings of difficult or worrisome behavior of young children at home or in the classroom. For example, externalizing behavior is often, but not necessarily, associated with attachment disorganization. When doll play suggests secure attachment yet there is evidence of behavior problems, it may be wise to consider other sources of these problem behaviors. However, we strongly recommend against the use of such assessments as a way of formally assessing the mental health of particular young children or the quality of family life.

The ADPA is a demonstrated valuable and valid tool to use to unravel children’s inner experience. It requires considerable training to administer and code reliably. Furthermore, classification information can have the unfortunate effect of labeling or pathologizing young children. Although attachment classifications appear to reflect different levels of developmental risk in a population, they do not convey specific diagnostic information for particular children. Considerably more research is required with respect to both the ADPA and other symbolic representational assessments to understand fully their predictive strengths and limitations.

## Author Contributions

CG and JS share equally the data set presented in this paper. CG was in charge of data analysis and was the primary author responsible for writing the paper. JS contributed ideas and editing to the paper.

## Conflict of Interest Statement

The authors declare that the research was conducted in the absence of any commercial or financial relationships that could be construed as a potential conflict of interest.
